# Supporting Future Cannabis Policy – Developing a Standard Joint Unit: A Brief Back-Casting Exercise

**DOI:** 10.3389/fpsyt.2021.675033

**Published:** 2021-05-20

**Authors:** Hugo López-Pelayo, Silvia Matrai, Mercè Balcells-Olivero, Eugènia Campeny, Fleur Braddick, Matthijs G. Bossong, Olga S. Cruz, Paolo Deluca, Geert Dom, Daniel Feingold, Tom P. Freeman, Pablo Guzman, Chandni Hindocha, Brian C. Kelly, Nienke Liebregts, Valentina Lorenzetti, Jakob Manthey, João Matias, Clara Oliveras, Maria Teresa Pons, Jürgen Rehm, Moritz Rosenkranz, Zoe Swithenbank, Luc van Deurse, Julian Vicente, Mike Vuolo, Marcin Wojnar, Antoni Gual

**Affiliations:** ^1^Institut Clínic de Neurociències, Psychiatry and Psychology Service, Grup Recerca Addiccions Clínic, Institut d'Investigacions Biomèdiques August Pi i Sunyer, Hospital Clínic de Barcelona, Barcelona, Spain; ^2^Department of Psychiatry, University Medical Center Utrecht Brain Center, Utrecht University, Utrecht, Netherlands; ^3^Social Sciences Department, Instituto Universitário da Maia (ISMAI), Maia, Portugal; ^4^University Interdisciplinary Research Centre for Human Rights - JusGov, University of Minho, Maia, Portugal and JusGov - Escola de Direito, Braga, Portugal; ^5^Addictions Department, King's College London, Institute of Psychiatry, Psychology & Neuroscience, London, United Kingdom; ^6^Adult Psychiatry Department, Collaborative Antwerp Psychiatric Research Institute, University of Antwerp, Antwerp, Belgium; ^7^European Federation of Addiction Societies, Boechout, Belgium; ^8^Department of Psychology, Ariel University, Ariel, Israel; ^9^Addiction and Mental Health Group, Department of Psychology, University of Bath, Bath, United Kingdom; ^10^Clinical Psychopharmacology Unit, Research Department of Clinical and Health Psychology, University College London, London, United Kingdom; ^11^National Institute for Health Research (NIHR) University College London Hospitals Biomedical Research Centre, University College Hospital, London, United Kingdom; ^12^Departament of Sociology, Purdue University, West Lafayette, IN, United States; ^13^Bonger Institute of Criminology, University of Amsterdam, Amsterdam, Netherlands; ^14^School of Behavioural and Health Sciences, Faculty of Health Sciences, Australian Catholic University, Fitzroy, VIC, Australia; ^15^Institute of Clinical Psychology and Psychotherapy, Technische Universität Dresden, Dresden, Germany; ^16^Department of Psychiatry and Psychotherapy, Center for Interdisciplinary Addiction Research of Hamburg University (ZIS), University Medical Center Hamburg-Eppendorf (UKE), Hamburg, Germany; ^17^European Monitoring Centre for Drugs and Drug Addiction, Lisbon, Portugal; ^18^Institute for Mental Health Policy Research, Centre for Addiction and Mental Health, Toronto, ON, Canada; ^19^Campbell Family Mental Health Research Institute, Centre for Addiction and Mental Health, Toronto, ON, Canada; ^20^Canada Epidemiological Research Unit, Canada Dalla Lana School of Public Health and Department of Psychiatry, University of Toronto (UofT), Toronto, ON, Canada; ^21^Technische Universität Dresden, Klinische Psychologie & Psychotherapie, Dresden, Germany; ^22^Department of International Health Projects, Institute for Leadership and Health Management, I.M.Sechenov First Moscow State Medical University, Moscow, Russia; ^23^Faculty of Health, Public Health Institute, Liverpool John Moores University, Liverpool, United Kingdom; ^24^Student Governance and Leadership in European Public Health, Maastricht University, Maastricht, Netherlands; ^25^Department of Sociology, The Ohio State University, Columbus, OH, United States; ^26^Department of Psychiatry, Medical University of Warsaw, Warsaw, Poland; ^27^Department of Psychiatry, University of Michigan, Ann Arbor, MI, United States

**Keywords:** cannabis, standard units, harm-reduction, risky use, prevention

## Abstract

The standardization of cannabis doses is a priority for research, policy-making, clinical and harm-reduction interventions and consumer security. Scientists have called for standard units of dosing for cannabis, similar to those used for alcohol. A Standard Joint Unit (SJU) would facilitate preventive and intervention models in ways similar to the Standard Drink (SD). Learning from the SD experiences allows researchers to tackle emerging barriers to the SJU by applying modern forecasting methods. During a workshop at the Lisbon Addictions Conference 2019, a back-casting foresight method was used to address challenges and achieve consensus in developing an SJU. Thirty-two professionals from 13 countries and 10 disciplines participated. Descriptive analysis of the workshop was carried out by the organizers and shared with the participants in order to suggest amendments. Several characteristics of the SJU were defined: (1) core values: easy-to use, universal, focused on THC, accurate, and accessible; (2) key challenges: sudden changes in patterns of use, heterogeneity of cannabis compounds as well as in administration routes, variations over time in THC concentrations, and of laws that regulate the legal status of recreational and medical cannabis use); and (3) facilitators: previous experience with standardized measurements, funding opportunities, multi-stakeholder support, high prevalence of cannabis users, and widespread changes in legislation. Participants also identified three initial steps for the implementation of a SJU by 2030: (1) Building a task-force to develop a consensus-based SJU; (2) Expanded available national-level data; (3) Linking SJU consumption to the concept of “risky use,” based on evidence of harms.

## Introduction

After tobacco and alcohol, cannabis is the most widely used psychoactive substance worldwide. Societies are experiencing a normalization of its use, especially among youth (1) as illustrated by the growing phenomena of coffeeshops and cannabis social clubs (2). Cannabis policy is shifting worldwide as the supply is moving from an unregulated (illicit) market to an open market for an “ordinary commodity” (e.g., in Canada, Uruguay and several states within the US). Observing that public opinion on the legal status of cannabis in Europe is also changing, European countries likely will not be an exception to this trend over the coming years. This changing context (i.e., in social perceptions and in legal context in some countries) aligns cannabis use in high-income countries more closely with alcohol or tobacco than to currently illegal drugs. A transition to legal, regulated access will require new prevention and harm-reduction strategies to minimize adverse effects as cannabis becomes more widely available (3). However, evidence also points to higher THC concentration in cannabis products during the last decade, which is believed to be associated with an increased risk of acute, and chronic health problems, especially in adolescents (4). Additionally, the National Institute on Drug Abuse has already expressed plans to “explore the possibility of constructing a standardized dose similar to that for alcohol (the standard drink) and tobacco (a cigarette) […for cannabis] for researchers to employ in analyzing use and […] for users to understand their consumption (5).” Learning from the history of measuring standard units, i.e., alcohol and tobacco, could facilitate public health, research and clinical professionals to navigate this new context more successfully and prevent errors from being repeated. During the 1980s and 1990s, several countries reached a national consensus defining their Standard Drink (SD) (6). Researchers conducted field tests in several countries to grow comparative evidence and adapt prevention efforts to the cultural characteristics of the country (7). However, most countries did not re-validate the SD with the field test (8). As a result, there are large differences between countries in defining SD, due to the fact that some are based on national consensus while others derive from experimental research, making useful cross-country comparison, policy analysis and prevention efforts more difficult. Nonetheless, despite its limited accuracy, the SD has advanced the alcohol public health field considerably: the SD provides clinicians, public health specialists, policy makers, and researchers with a common tool for assessing alcohol use and implementing programs from early identification of risky use (9) to monitoring consumption in harm-reduction (10).

Other relevant instruments for assessing alcohol use were based on the SD [AUDIT (11), ISCA (12), AUDIT-C (13), HRAR (14)] and are widely implemented globally. Screening and Brief Interventions (SBI) programs, make use of these instruments, are cost-effective in 24 out of 28 EU countries and cost-saving in 50% of countries (15). Learning from practical experiences in the alcohol field and the development and use of SD, the following should be essential characteristics in developing a Standard Joint Unit (SJU): (1) a high degree of evidence-based consensus on equivalence between countries; (2) high accuracy (providing a faithful representation of real doses); (3) taking into account less common routes of administration (cannabis is consumed in more varied ways than alcohol or tobacco); (4) built in monitoring of changes in patterns of use and chemical composition. Having said this, many peculiarities of cannabis use present challenges in the development of standard units for cannabis, among these are: different routes of administration (smoking, vaping, edible), concurrent use with other substances (e.g., tobacco, alcohol), heterogeneity of quantities or interactions among different cannabinoids (THC/CBD) (16). Standard units for cannabis, based on a fixed dose of THC, have the potential to address some of these challenges (16). What constitutes a SJU is important to consider. Currently, studies have gathered evidence on typical joints in Australia (140 mg cannabis/joint), Spain (250 mg of hashish or cannabis plant/joint and translating into 7 mg THC/joint), The Netherlands (260 mg cannabis/joint), UK (140 mg cannabis/joint and 380 mg cannabis/joint), USA (660 mg cannabis/joint vs. 580 mg cannabis/joint vs. 700 mg cannabis/joint) (17–24). Only the Spanish study reported milligrams of THC in a typical joint. Although a commendable start, these studies were heterogeneous regarding both methods (real/simulated cannabis, ecological/lab studies, etc…) and results, even within countries. In the European Web Survey on Drugs (25), the EMCDDA also asks about usual amount consumed for herbal cannabis and cannabis resin. The rapid growth of research in this field also means that reaching a consensus on SJU research methodologies to support clinical implementation is an urgent issue. In order to advance this area, we organized a workshop, as part of the Lisbon Addictions Conference 2019, with experts in different disciplines (sociology, psychology, public health, basic and clinical research, psychiatry) and with the following objectives: (1) to reflect on the challenges to reaching a consensus on an operative SJU; (2) to reflect on opportunities and facilitators to achieving an SJU; (3) to propose different trajectories to achieve the main goal: implementation of a European SJU by the year 2030; and (4) to reach a minimum-level consensus on the first step toward achieving a SJU. The expected outputs were: (1) consensus on the first-steps toward achieving an SJU; and (2) a preliminary annual roadmap to develop a SJU by the year 2030.

## Methods

### The Back-Casting Exercise (BCE)

An operational definition of a BCE is “a scenario technique where normative targets or unwanted outcomes are defined by a group for the purpose of formulating ways in which such goals can be achieved or avoided” (26). Participants in back-casting exercises do not predict the future, but rather choose the desirable future and work backwards to define the steps to achieve that goal (26). Back-casting is a prospective method in the context of foresight methodologies. Foresight methodologies are “frameworks for making sense of data generated by structured processes to think about the future” (27). A back-casting exercise is useful when (28, 29):

1. the problem is complex, persistent, and predominant.
2. change is very necessary.
3. sustainability of the solution is relevant.
4. long-term planning (at least 5 years) is needed.
5. the results of the exercise could impact multiple stakeholders and could empower the participants in the exercise.

**Figure 1:**
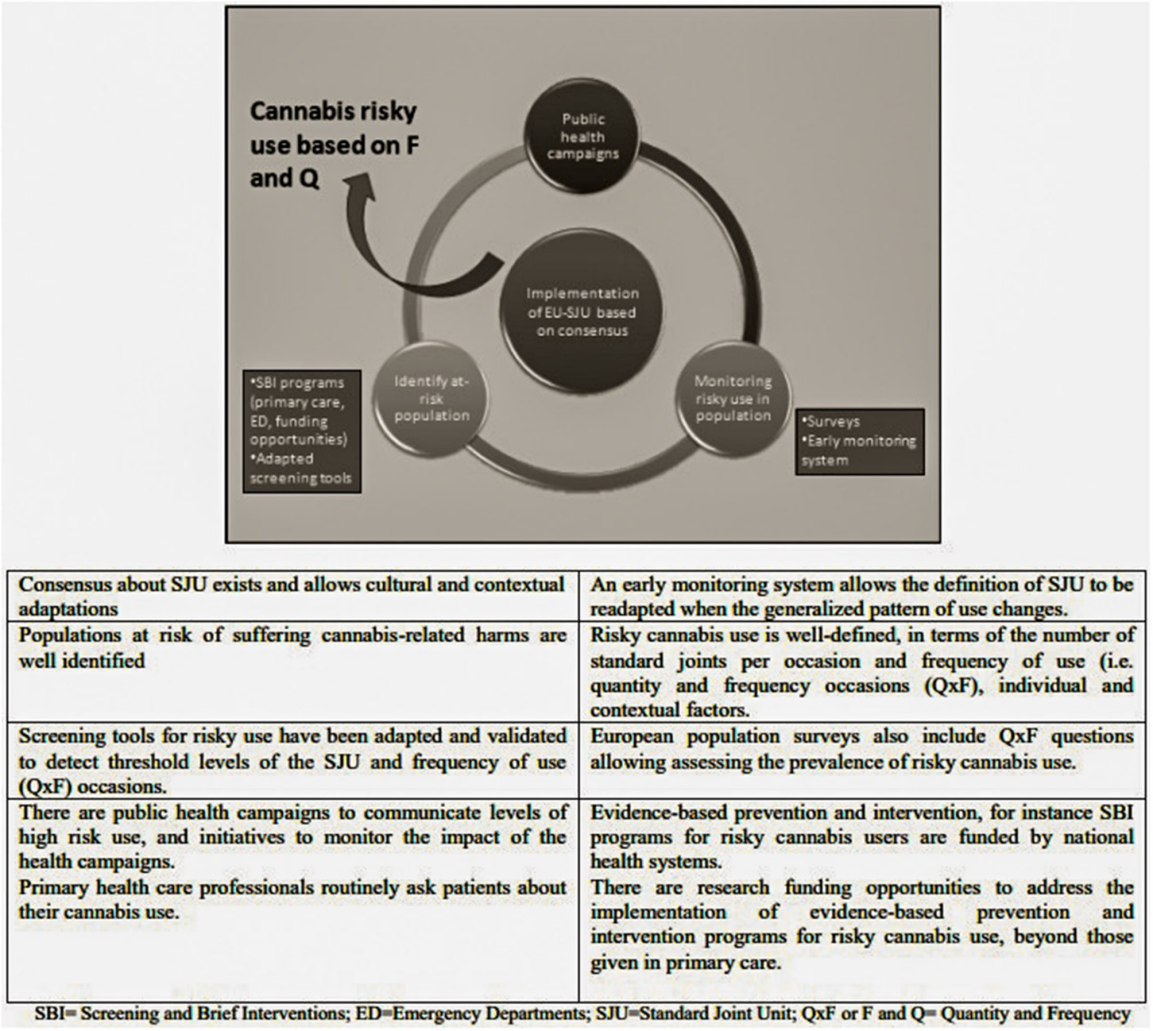
Hypothetical desirable future in 2030, used to guide the back-casting workshop.

The organizers pre-defined the desirable future in 2030 based on their professional expertise in the alcohol and cannabis areas (see Figure 1). The contrast between desirable future and current scenario (see below) is the starting point for the workshop discussions. The current scenario was defined as:

- The populations at risk of suffering cannabis-related health problems are not well-identified.
- The assessment of cannabis use patterns is usually based on frequency of use (e.g., days) only.
- A clear public health message about “how much is too much” does not exist because low-risk use is not well-defined.
- The prevalence of risky use (in different populations) is unknown due to lack of risk level definitions.
- Evidence-based practices to reduce cannabis-attributable harms (i.e., SBIRT) are not implemented.

### Participants

**Figure 2:**
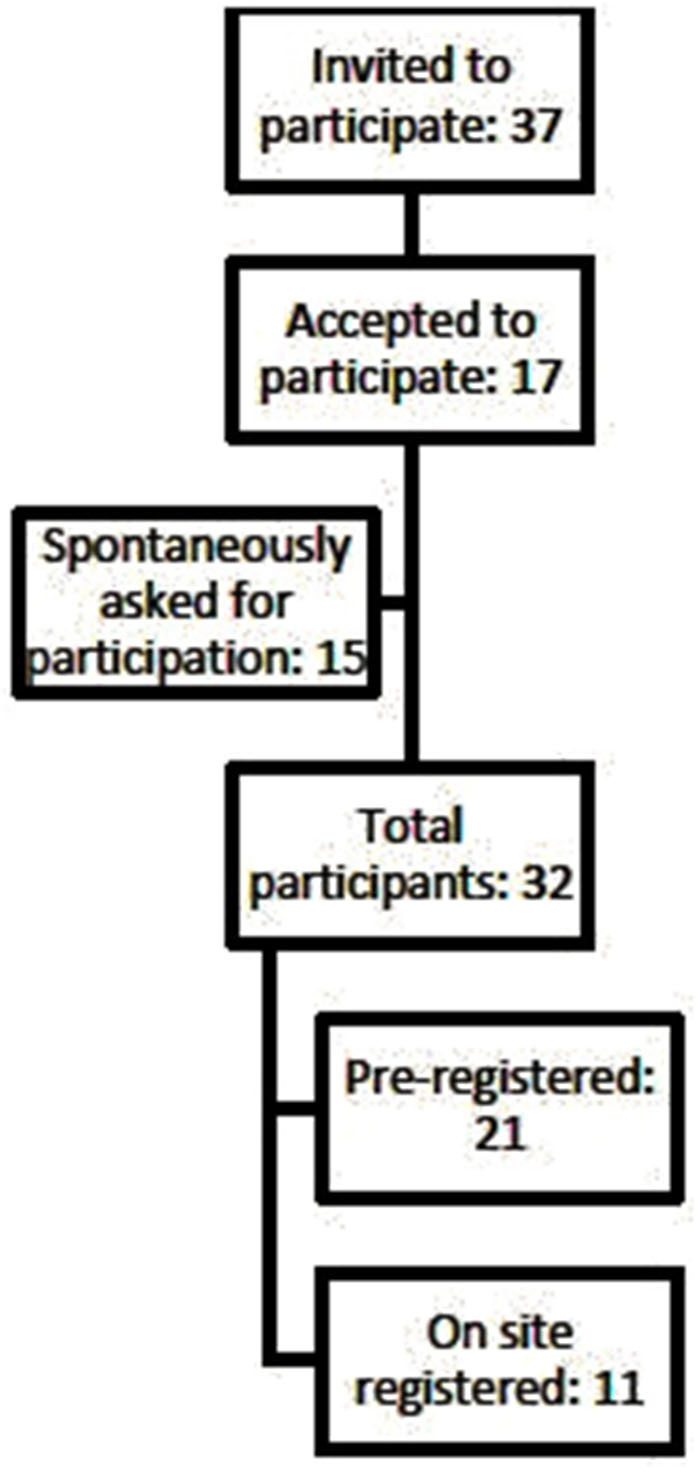
Recruitment process of participants in the workshop.

A total of thirty-two experts attended the workshop. Participants were scholars and practitioners from a range of disciplines: basic research (*n* = 1), pharmacology (*n* = 1), neuroimaging (*n* = 3), social sciences (*n* = 3), psychology (*n* = 3) and other clinical research (*n* = 10), public health (*n* = 4), epidemiology (*n* = 4), law and criminology (*n* = 2). Furthermore, one cannabis industry representative participated. Experts were divided into five transdisciplinary groups. Participants came from several different countries (in descending order of number): UK (*n* = 7), Spain (*n* = 5), Portugal (*n* = 5), The Netherlands (*n* = 3), USA (*n* = 2), Germany (*n* = 2), Australia (*n* = 2), Belgium (*n* = 1), Hungary (*n* = 1), Poland (*n* = 1), Cyprus (*n* = 1), Israel (*n* = 1) and Canada (*n* = 1). Participants had either pre-registered for the back-casting workshop (*n* = 21) or arrived to participate spontaneously (*n* = 11) (these participants were admitted until all available seats were occupied). The workshop was comprised of both academics invited, based on their expertise (*n* = 17); and participants from the conference (*n* = 15) (see Figure 2).

In order to facilitate the workshop dynamics and facilitate a smoother running of the exercise, those who had previously registered received a 3-page background document on the SJU concept, the back-casting method, and relevant key references, along with the following advice: (1) An absolute consensus is not expected. Please focus on achieving minimum consensus; (2) Try to find cause-effect relationships; (3) Try to focus on one future desirable scenario; and (4) Do not attempt to predict the future but rather consider the desirable future. The exercise was led by two clinician scientists, both with extensive experience in participatory workshops (AG and HLP). Three researchers – two of them with ample experience in participatory processes – collaborated in the design, preparation, and deployment of the workshop, and the analyses of the results (SM, EC and FB). These five experts conceptualized, designed and developed the exercise.

### Procedure (90 min)

We prepared and set up the back-casting exercise in the following steps (adapted from “STD back-back casting approach” and Wilson et al. 2006) (30):

Step 1 (10 min): Introduction - The first part of the session was dedicated to explain the rationale underlying the workshop, its objectives, methodology and expected outcomes. Afterwards, a description of the current scenario and a future desirable scenario was presented to the participants, with sufficient time reserved for questions or amendments to both current and future scenarios.
Step 2 (20 min): Prioritizing relevant elements - Activity 1 was explained and participants were allocated to small multi-disciplinary groups (6–7 people) for the first part. They had three lists of elements referring to the SJU: (i) challenges, (ii) facilitators, (iii) values (see Supplementary Tables 1–3). The lists included a definition for each concept. Participants could propose new items if they considered that the definition was not accurate or if a concept was missing. Each small group was instructed to choose by consensus the five most relevant concepts from each list. In the second part of the exercise the whole workshop group worked together and voted on each concept for relevance, after hearing the outcomes of previous consensus discussions.
Step 3 (30 min): Back-cast trajectories - Activity 2 from future to present was performed by each small group (*n* = 5, of 6–7 participants each). The groups focused on a specific key element of the bigger desirable future scenario, and each had a card with the description of this element (group 1: Primary care; group 2: Prevention; group 3: Cannabis users; group 4: Epidemiology; group 5: Research). Using a pre-designed canvas, each group deconstructed the route toward the end-point of the specific scenario element in 2030, starting in 2020 (see Supplementary Table 4). At the end of this exercise, the results were briefly shared with the other members of the workshop.
Step 4 (10 min): Defining key events - this slot was allocated to a discussion across the groups of cornerstones, milestones, and first steps based on the reflection during the exercise and the professional background of the participants.

Summary (5 min): The exercise ended with a brief summary given by one of the participants (TPF), as rapporteur of the group. The participant was one of the coordinators of the preconference workshop “International Cannabis Toolkit,” in order to link these events. (https://www.lisbonaddictions.eu/lisbon-addictions-2019/side-events). The context of the FuturiZe Project and the Lisbon Addictions Conference is explained in the Supplementary Material.

### Analyses

Descriptive analysis of the workshop was carried out by the organizers and shared with the participants in order to suggest amendments. No quantitative or qualitative analyses were conducted.

Ethical issues: Under Spanish law, no ethical approval was required for this study in which the data is expert opinion.

## Results

### Future Desirable Scenario

The workshop participants did not raise any modifying comments or objections on the desirable future scenario (i.e., implementation of a SJU based on consensus) as proposed by the organizers, and approved unanimously it (see Figure 1).

### Defining Values, Challenges and Facilitators (Supplementary Tables 1–3 Respectively)

The five most highly voted defining values associated with SJU were “easy-to-use” (straightforward, clear instructions and simple to use correctly, 100%), “universal” (appropriate for or adjustable to all settings/contexts, 100%), “accounts for THC” (quantity of use register will only include THC, 80%), “accurate” (providing a faithful representation of someone or something, 60%) and “accessible” (easily understood or appreciated, 60%). The five most highly voted challenges were: “sudden changes in patterns of use” (quick and unexpected changes in the behavior of cannabis users which impact the validity/accuracy of the SJU, 100%), “heterogeneity of cannabis compounds” (diversity in content/composition, 80%), “heterogeneity of THC concentration” (diversity on THC content for the same grams of herbal or resin, 80%), “heterogeneity in routes of administration” (diversity in routes of administration (smoking, vaping, edible, etc.), 60%) and “laws” (legal status of marijuana (e.g., possession being criminal offense) in many countries, 60%). “Synthetic cannabinoids” were proposed as a separate additional challenge by one participant, but this challenge was included by consensus of participants in the category of “heterogeneity of cannabis compounds.” The six most highly voted facilitators were: “previous experience in other standard measurements” (Learning about the limitations and strengths of standardization of typical dose and operational definitions of risky use in tobacco or alcohol, 100%),” funding opportunities available” (money provided, especially by an organization or government, for drug research is now addressed to the area of cannabis, 80%), “cannabis users' support” (organized or non-organized users whose messages are partially or totally in line with the objectives of the SJU 80%), “policy-makers' support” (roadmap or agenda of policy-makers is partially or totally in line with the objectives of SJU, 60%), “high prevalence of use” (health topic becomes more prevalent and more mainstream,60%), “depenalization, decriminalization and legalization in many countries” (changes in laws regarding cannabis which facilitate research into cannabis and the implementation of solutions conducive to harm-reduction approaches, 60%). “New advances in laboratory studies” were proposed and accepted as an additional facilitator, which was voted on by a majority of the groups (60%).

### Back-Casting Trajectories (From 2030 to 2020) and Milestones (Supplementary Table 4)

The most salient milestones reported by participants were: (1) negotiate and engage the stakeholders as an ongoing process; (2) set of scenarios (options) to discuss the analytical phase; (3) guidelines for using the SJU (setting, protocols, etc.); (4) definition and consensus of SJU [and conversion to standard cannabis unit (16)]; (5) programs funding EU-wide research in the cannabis field; (6) external validation (statistical concept) of SJU (e.g., indicators) before clinical programs; and (7) data collection (dose per joint) at the country level. Consideration of whether it is inappropriate (e.g., normalization of drug-using behavior and reduced perception of risk) or appropriate (e.g., reducing stigma and increasing help-seeking) to use the term “standard” when it comes to a substance that is illegal in many jurisdictions also arose as relevant point during the workshop process.

### First Steps

The first three steps (to be implemented concurrently) were: (1) Set up a “Task Force” that could also act as a lobby for the European Commission and influence the European Union (EU) Research Agenda, raising the profile of this subject; (2) Conduct a review of already available data at the national level; (3) Emphasize the need for SJU in terms of risks.

## Discussion

The 21st century has been characterized as an “Information Age,” where technologies facilitate the use of information by citizens. The SJU provides an opportunity to capitalize on this desire for information by working toward a clear evidence-based standard which consumers can rely upon. In addition to leveraging consumer desires for information, the SJU provides important opportunities for harm reduction and intervention as the use of cannabis continues to expand in the future. In fact, although our proposal of establishing a SJU is mainly focused on regulation of recreational use, it might also be useful to achieve a better control of those preparations intended for a potential medical use, which are also generating growing interest in the last years (31). Given the importance of these standards for the future of cannabis consumption, the process of identifying the most efficient and accurate means to develop the SJU remains a critical task. The results described above used established expertise across multiple scientific domains to identify how these standards may be achieved. According to the expert opinion from the workshop group, the SJU must be easy-to-use, universal, take into account only the concentration of THC, and be accurate, and accessible (“easily understood or appreciated”). With the aim of overcoming the barriers identified and enhancing the effect of the facilitators, the experts suggested one main step to be implemented: creating a task force to emphasize the need for the development of an SJU. This task force should generate input for the EU Research Agenda and promote a review of the available data at the national level. The majority of defining values reported by the participants were also presented in two recent opinion papers (e.g., assessing only THC, accessible, universal and easy-to-use) (16, 32). The SJU should be accurate (defined as “providing a faithful representation of something”) according to attendees, being different to the SD, which prioritized utility over accuracy (6). Most of the challenges discussed [i.e., heterogeneity of routes of administration, laws, variations over time in THC concentrations (33), compounds and patterns of use] have also been repeatedly reported as limitations in previous research (16, 17). Future research must cope with these barriers by incorporating new methods [e.g., trend-spotter method (34), foresight methods (27), participatory research (35), etc.]. An SJU Task Force should share the necessary knowledge, skills and expertise in such new methods. The current legal status of cannabis in 12 European countries is more flexible now than it was a few years ago (e.g., incarceration is now not possible for minor cannabis possession in these 12 countries) and continues to change (e.g., the government of Luxembourg is set to provide legal access to cannabis in the near future). These evolutions in policy could easily open up more research opportunities in this area (36, 37). Cannabis is high both on the research and regulatory agenda – a PubMed search using the terms “marijuana OR marihuana OR cannabis” showed 388 papers in 1998 and 2,190 papers in 2018, thus research interest, measured by published papers, has increased by 460% within two decades. Over the same period, the increase of the number of papers studying “cocaine” was only 8.2%. These patterns reflect a growing interest in this research area, an interest that might act as a facilitator for establishing a SJU research agenda, making it important for researchers to use this momentum to promote the specific line of research on the SJU. Increased funding opportunities in Australia, North America, and Europe are beginning to facilitate much needed research to establish the SJU. The National Focal Point in Spain for the EMCDDA (Plan Nacional sobre Drogas) funded two projects related to the SJU. NIDA also funded research for screening and brief assessment, development and impact assessment of prevention programs on marijuana use and patterns and trends in marijuana use and attitudes (38). These topics are closely related to the development of an SJU. In 2015, NIDA invested US$ 66M in cannabis research [> 10% of all research project grants, US$ 625M (39)]. In Europe several opportunities exist, for example: Supporting Initiatives in the Field of Drugs Policy (JUST-DRUGS-AG HOME Action Grant) and European Cooperation in Science and Technology (40, 41). Recently, NIDA launched a request for information inviting Comments on the Establishment and Implementation of a Standard Unit Dose of Δ-9-tetrahydrocannabinol (THC) for Cannabis Research (42). Moreover, the fact that cannabis is the most widely used psychoactive substance beyond alcohol and tobacco, with some authors even claiming that there is a certain normalization of its use, should also stimulate research in order to overcome the gaps in the specialized literature. Taking advantage of funding opportunities was critical, according the expert opinion of participants, in order to enable the creation of a task force that allows oversight of the available data at the national levels, and to act as lobbying force to influence a cannabis research agenda. This network could both facilitate research and be involved in training relevant workforces in use of the standard measures.

### Limitations and Strengths

The main limitation to our workshop back-casting exercise was a time constraint (90 minutes vs. 4 or more hours for other published BCEs), which may have resulted in less intermediate analyses between description and consensus of desirable futures (step 1), and back-cast trajectories (step 2) (43). However, this brief and concentrated version of BCE allows for the inclusion of a large number of diverse experts who otherwise would not have been able to attend for timetabling or financial reasons. Another secondary limitation is the limited heterogeneity of participants (with few from outside academia). Fortunately, we think that synergies with other activities in the LxAddiction2019 Conference will have mitigated these limitations (e.g., a preconference Workshop on 'International Cannabis Toolkit' https://canntoolkit.com/ and a “Big Debate” session on Day 1 of the conference programme: 'Will changes in cannabis policy result in greater costs or greater benefits?'). The strengths of this BCE exercise were the relevant expertise in this specific research area of the vast majority of participants; heterogeneity of research profiles involved (basic science, social science, epidemiology, neuroimaging, pharmacology, clinical research) and the inspiring context of the FuturiZe Project and conference which facilitated creativity and the opportunity for participants to engage in a co-creative exercise.

## Conclusions

The implementation of a SJU in 2030 was considered feasible after overcoming several barriers and harnessing contextual facilitators. Experts agreed that an SJU is possible on the basis on the following achievements: (1) the building of a task force to define, develop and advocate for an evidence-based SJU; (2) reviewing and expanding available national-level data on cannabis use and related risks; and (3) examining how the SJU relates to the concept of “risky use” of cannabis.

## Data Availability Statement

The raw data supporting the conclusions of this article will be made available by the authors, without undue reservation.

## Ethics Statement

Ethical review and approval was not required for the study on human participants in accordance with the local legislation and institutional requirements. Written informed consent for participation was not required for this study in accordance with the national legislation and the institutional requirements.

## Author Contributions

HL-P, AG, SM, EC and FB designed the workshop and the study (conceptualization), and conducted the analyses. HL-P wrote the first draft (writing original draft). All authors contributed to the article and approved the submitted version.

## Conflict of Interest

One of the participants in the workshop was a representative of cannabis industry (not an author of this paper). CH is employed by GW Pharmaceuticals. Her substantive contribution to this publication occurred before employment at GW pharmaceuticals. The remaining authors declare that the research was conducted in the absence of any commercial or financial relationships that could be construed as a potential conflict of interest.
