# Supplementary material for: Supporting Future Cannabis Policy – Developing a Standard Joint Unit: A Brief Back-Casting Exercise
Source: Front Psychiatry. 2021 May 20;12:675033. doi: 10.3389/fpsyt.2021.675033 (PMC8172797; doi:10.3389/fpsyt.2021.675033)
Supplement: Supplementary file 1 [file Data_Sheet_1.pdf]

Table 1. List of values including their definition and received group votes.

Values		Definition	Group votes (n=5)*	% of votes
1	Flexible	Can be easily modified to respond to diverse and changing circumstances	2	40
2	Easy-to-use	Straightforward, clear instructions and simple to use correctly	5	100
3	Accurate	Providing a faithful representation of someone or something	3	60
4	Quick	Lasting or taking a short time (to use)	0	0
5	Universal	Appropriate for or adjustable to all settings/contexts	5	100
6a	Accessible –definition 1 -	Can be easily obtained for use	2	40
6b	Accessible – definition 2 -	Easily understood or appreciated	3	60
7	Easy to update	Gives (and incorporates) the latest information about something without excessive additional effort	0	0
8	Take into account both THC and CBD	Quantity of use register will include both THC and CBD	1	20
9	Take into account only THC	Quantity of use register will only include THC	4	80
10	Culturally adapted	Become adjusted to the ideas, customs, and social behaviour of different societies or social groups	0	0
11	Other. Specify (even if it is a slight variation of one of the previous values)	None	N/A	N/A

THC= Tetrahydrocannabinol ; CBD= Cannabidiol; N/A= Not applicable

Table 2. List of challenges including their definition and received group votes.

Challenges		Definition	Group votes (n=5)*	% of votes
1	Heterogeneity of cannabis compounds	Diversity in content/composition (quantities/proportions of THC, CBD, etc.)	4	80
2	Heterogeneity of RoAs	Diversity in Routes of Administration (smoking, vaping, edible, etc.)	1	20
3	Individual factors (e.g. mental health)	Individual factors determine or modulate pattern of use and risks	1	20
4	Concomitant use of other drugs (e.g. tobacco, NPS, alcohol)	Use of other drugs at the same time as cannabis use is a confounding factor for patterns of use and risks	1	20
5	Funding restrictions	Money provided, especially by an organization or government, for cannabis research is limited	0	0
6	Heterogeneity of THC concentration	Diversity on THC content for the same grams of herbal or resin	4	80
7	Laws	Legal status of marijuana (e.g. possession being criminal offense) in many countries which could act as barriers in research and implementation of the SJU	3	60
8	Influence of industry	Roadmap or agenda of industry actors (for both medical use and recreational use) which can have an impact against the objectives of SJU	3	60
9	Influence of civil society	Civil society organisations or actors working in the area of cannabis or other drugs can have a negative impact on research and implementation of SJU(e.g. Those lobbying for decriminalisation might be against considering cannabis as a health risk)	1	20
10	Sudden changes in the pattern of use (e.g. vaping)	Quick and unexpected changes in the behaviour of cannabis users which impact the validity/accuracy of the SJU (e.g. making it out of date)	5	100
11	Other. Specify (even if it is a slight variation of one of the previous values)	None	N/A	N/A

RoAs= Routes of Administration; NPS= Novel Psychoactive Substances; THC= Tetrahydrocannabinol ; CBD= Cannabidiol; SJU=Standard Joint Unit; N/A= Not applicable

Table 3. List of facilitators including their definition and received group votes.

Facilitators		Definition	Group votes (n=5)*	% of votes
1	Depenalisation, decriminalisation and legalisation of cannabis use in many countries	Changes in laws regarding cannabis which facilitate research into cannabis and the implementation of solutions conducive to harm-reduction approaches	3	60
2	Reduction in stigma of cannabis users	In recent years, attitudes and beliefs that lead people to reject, avoid, or fear those who use cannabis have decreased	0	0
3	Previous experience in other standard measurements (e.g. Standard Drink Units)	Learning about the limitations and strengths of standardisation of typical dose and operational definitions of risky use in other substances (e.g. SDUs on alcohol, package/year on tobacco).<br>This should lead us to develop better ways to establish consensus around an SJU.	5	100
4	Industry support	Roadmap or agenda of industry actors (for both medical use and recreational use) which is partially or totally in line with the objectives of SJU, and supports the need to define and establish a criteria of risky use based on SJU	0	0
5	Users support	Organised (lobbies) or non-organised (influencers) users whose messages are partially or totally in line with the objectives of the SJU, and support the need to define and establish a criteria of risky use based on an SJU	4	80
6	Civil society support	Society (as a whole) is mostly in favour of the objectives of SJU, and supports the need to define and establish a criteria of risky use based on an SJU	0	0
7	Policy-makers' support	Roadmap or agenda of policy-makers is partially or totally in line with the objectives of SJU, and supports the need to define and establish a criteria of risky use based on an SJU	3	60
8	Momentum	The impetus and driving force gained by the course of events (politically, in media, etc.) in the last year is internationally and globally (policy, civil society, industry, media, popular culture) in favour of developing an SJU	1	20
9	High prevalence of use	When a health topic becomes more prevalent and more mainstream (affecting, or known about by a greater proportion people and regarded as normal or common) it can facilitate research	3	60
10	Funding opportunities available	Money provided, especially by an organization or government, for drug research is now addressed to the area of cannabis	4	80
11	Other. Specify (even if it is a slight variation of one of the previous values)	New advances in laboratory studies	3	60

SJU= Standard Joint Unit; SDU= Standard Drink Unit

Table 4. Scenarios and trajectories of back-casting exercise.

Scenarios	2030	2029	2028	2027	2026	2025	2024	2023	2022	2021	2020	2019
1: Primary care	Implementation of BI with risky use (based on SJU)		Training GP	Guidelines accepted by professional associations	Randomized Controlled Trials of Brief Interventions (IBs) for cannabis risky use	Develop manning for BI	Funding for clinical trials	Validation of tools based on SJU		Development of tools based on SJU Funding for the development of tools	Consensus on SJU	
2:Prevention	Deliver	Co-production	Implication & civil society involvement to tailor messages &perceptions	Test campaign	Government support for campaign	Development of campaign	Consolidate evidence to inform next step	Critical research & data collection. Multiple countries where cannabis is legal & illegal "frequency & SJU". Track users over time &rely on existing population surveys	At risk population data	Consensus on SJU	Map reality (issues) & opportunities. Funding (ongoing)	Funding opportunities Stakeholders + strategic planning: research, public health, users, policy makers, pharmacological producers
3: Cannabis user	Cannabis online test + referral to general practitioner or specialist	Increased capacity in health care system to treat high numbers of users (potential) + (funding)		Online screening test	Update according the changes in cannabis potency	Increased public knowledge of SJU	Funding: Randomized Controlled Trials in Primary Care	Funding: Feasibility of harm reduction – reduction of SJU use. Development of suitable intervention user's understanding of SJU.		Funding: Development of screening test + validation. Links between SJU and harm.	Research program across EU. Establish SJU (dose)	Funding
4: Epidemiology	Implementation	Dissemination to professionals		Refinement of measure		Validation of pilot tests of SJU		Consensus of definition of SJU		Research on patterns of use across countries	Consortium development	

5: Research	All researchers: knowledge SJU	Follow-up	Implementation phase	Formalize policy	Final consensus meeting main execution	Validation	Analyze data 1st evaluation	Start a pilot	Design of a pilot	Needs and requirements Assessment Stakeholders	Agenda for development	Designing the scenarios

BI= brief intervention; GP= General Practitioner; SJU= Standard Joint Unit; EU= European Union

Supplementary material: ***The context of FuturiZe Project and Lisbon Addictions Conference***

The workshop was developed in the context of a FuturiZe project (http://www.lx-futurize.eu) FuturiZe is a European project, co-funded by DG JUST at the European Commission, which runs from October 2018 to March 2020, and which co-produced a strand of the 3rd European Conference on addictive behaviours and dependencies **(LxAddictions19, 23-25 October 2019)**, on ‘the futures of addiction’. The project aimed to provide a space for EU-wide, multi-stakeholder and inter-sectoral networking and structured interactive debates, open to all conference delegates, focusing on key future scenarios in the field of drugs and behavioural addictions. The objectives of the debates were to boost knowledge exchange and collaboration among the primary stakeholder sectors, and to enhance and support future-oriented policy thinking in the addictions areas.

The project has also set up an Addictions Foresight Network, launched at Lisbon Addictions 2019, with the aim of sustaining and extending the process started with FuturiZe. The partners of FuturiZe are Serviço de Intervenção nos Comportamentos Aditivos e nas Dependências (SICAD, General Directorate for Intervention on Addictive Behaviours and Dependencies), Hospital Clínic de Barcelona, Fundació Clínic per la Recerca Biomèdica and the European Federation of Addiction Societies (EUFAS). The activities of the FuturiZe Project were coordinated with the organizers of LxAddictions19 (SICAD and EMCDDA).
